# Cohort Profile: The DynaHEALTH consortium – a European consortium for a life-course bio-psychosocial model of healthy ageing of glucose homeostasis

**DOI:** 10.1093/ije/dyz056

**Published:** 2019-04-10

**Authors:** Sylvain Sebert, Estelle Lowry, Nicole Aumüller, Mercedes G Bermúdez, Lise G Bjerregaard, Susanne R de Rooij, Maneka De Silva, Hanan El Marroun, Nadine Hummel, Teija Juola, Giacomo Mason, Daniela Much, Elena Oliveros, Stavros Poupakis, Nina Rautio, Phillipp Schwarzfischer, Evangelia Tzala, Olaf Uhl, Cornelieke van de Beek, Florianne Vehmeijer, Juan Verdejo-Román, Niko Wasenius, Claire Webster, Leena Ala-Mursula, Karl-Heinz Herzig, Sirkka Keinänen-Kiukaanniemi, Jouko Miettunen, Jennifer L Baker, Cristina Campoy, Gabriella Conti, Johan G Eriksson, Sandra Hummel, Vincent Jaddoe, Berthold Koletzko, Alex Lewin, Maria Rodriguez-Palermo, Tessa Roseboom, Ricardo Rueda, Jayne Evans, Janine F Felix, Inga Prokopenko, Thorkild I A Sørensen, Marjo-Riitta Järvelin

**Affiliations:** 1 Centre for Life Course Health Research, Faculty of Medicine, University of Oulu, Finland; 2 Biocenter Oulu, University of Oulu, Finland; 3 Department of Genomics of Complex Diseases, School of Public Health, Imperial College London, UK; 4 Division of Metabolic and Nutritional Medicine, Dr. von Hauner Children’s Hospital, Ludwig-Maximilians Universität München, Munich, Germany; 5 Department of Paediatrics, School of Medicine, University of Granada, Spain; 6 Center for Clinical Research and Prevention, Bispebjerg and Frederiksberg Hospital, The Capital Region, Copenhagen, Denmark; 7 Department of Clinical Epidemiology, Biostatistics & Bio informatics, Amsterdam University Medical Centre); 8 Department of Epidemiology and Biostatistics, MRC-PHE Centre for Environment and Health, School of Public Health, Imperial College London, UK; 9 Generation R Study Group, Department of Pediatrics, Department of Child & Adolescent Psychiatry, Erasmus University Medical Center, Rotterdam, the Netherlands; 10 Institute of Diabetes Research, Helmholtz Zentrum München, and Forschergruppe Diabetes, Klinikum rechts der Isar, Technische Universität München, Neuherberg, Germany; 11 University College London, UK; 12 Abbott Nutrition, Spain; 13 Department of Obstetrics & Gynaecology, Amsterdam University Medical Centers, The Netherlands; 14 Generation R Study Group, Department of Epidemiology, Erasmus University Medical Center, Rotterdam, the Netherlands; 15 Mind, Brain and Behavior Research Centre (CIMCYC), University of Granada, Spain; 16 Department of Experimental Psychology, Psychological Processes and Speech Therapy, Universidad Complutense de Madrid; 17 Department of General Practice and Primary Health Care, University of Helsinki, Helsinki, Finland; 18 Folkhälsan Research Center, Helsinki, Finland; 19 Beta Technology, UK; 20 Research Unit of Biomedicine, Department of Physiology & Biocenter of Oulu, University of Oulu, Oulu, Finland; 21 Department of Gastroenterology and Metabolism, Poznan University of Medical Sciences, Poznan, Poland; 22 Medical Research Center Oulu, Oulu University Hospital and University of Oulu, Finland; 23 NovoNordisk Foundation Centre for Basic Metabolic Research, Section of Metabolic Genetics, Faculty of Health and Medical Sciences, University of Copenhagen, Denmark; 24 National Institute for Health and Welfare, Finland; 25 Generation R Study Group, Department of Pediatrics, Department of Epidemiology, Erasmus University Medical Center, Rotterdam, the Netherlands; 26 Harvard Medical School, USA; 27 Department of Medical Statistics, London School of Hygiene and Tropical Medicine, UK; 28 Laboratorios Ordesa, Spain; 29 Academic Medical Centre, The Netherlands; 30 Department of Public Health, Section of Epidemiology, Faculty of Health and Medical Sciences, University of Copenhagen, Denmark; 31 Department of Life Sciences, College of Health and Life Sciences, Brunel University London, UK

## Why was the collaboration set up?

**Figure 1.**
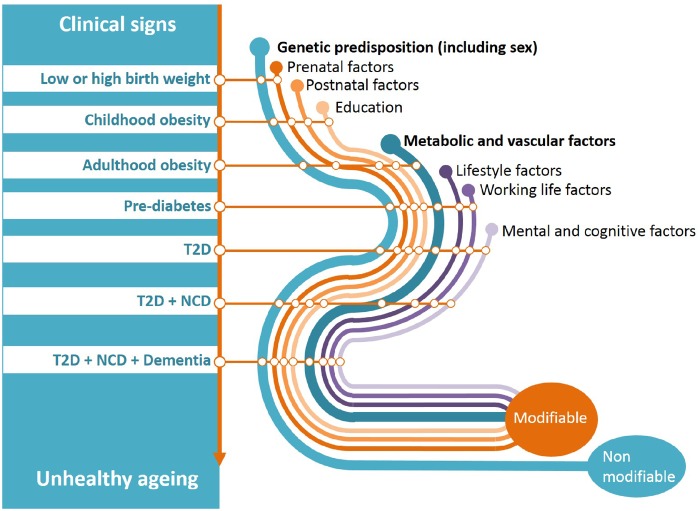
Illustration of the conceptual framework for unhealthy ageing from early life to old age. From a life-course perspective, it suggests that clinical events accumulate or lead to each other, and co-occur with factors that either can or cannot be modified. (T2D=type 2 diabetes, NCD=non-communicable disease). (Reproduced from https://www.dynahealth.eu/ with permission).

DynaHEALTH is a research consortium funded by the European Commission through the Horizon 2020 research programme. It was established to help solve the societal challenge of an ageing population and the associated burden of non-communicable diseases related to obesity and type 2 diabetes (T2D). The consortium brings together European researchers and datasets to build an empirical model of unhealthy ageing, with a longitudinal perspective, in which causal, bi-directional, mediating and confounding factors operate at a multi-dimensional level (Figure 1).

**Figure 2.**
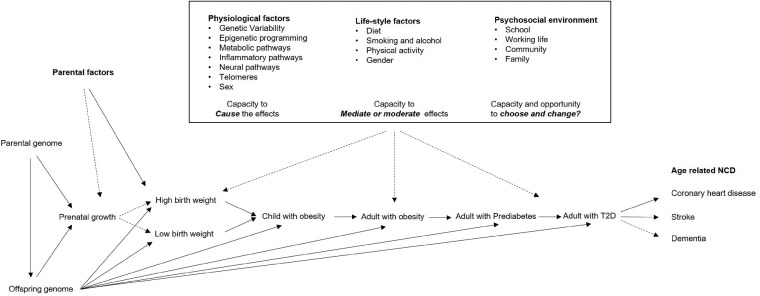
Physiological, lifestyle and psychosocial factors and stressors acting on life-course pathways linking altered adiposity in early life to non-communicable diseases at older ages.

The unhealthy ageing pathways often link altered adiposity in early life, early-onset obesity, T2D and the further accumulation of other chronic physical and mental non-communicable diseases in older ages. At each stage of the life-course there is potential to intervene either on suspected biological causes via classical clinical approaches, or other plausible causative pathways^1^ via integrated public health interventions (Figure 1). Successful interventions in early life have the potential to reduce subsequent investments in later life. Scientific collaborations in epidemiology and public health such as DynaHEALTH are built on a long-standing tradition of collecting prospective data. Moving on to the era of open sciences policy,^2^ meta-data analysis, the FAIR data principle (Findable, Accessible, Interoperable and Reusable) and the General Data Protection Regulation (GDPR), we must direct this public legacy in such a way as to better inform both policy makers and practitioners on complex patient and public health issues. Essentially, we must operate data to move beyond association studies and explore how the psychosocial factors, usually classified as confounders in medicine (Figure 2), can be analysed to help their operationalization and integration into healthcare programmes. Despite no apparent consensus in the literature on a single definition of psychosocial health, DynaHEALTH is referring to the following WHO definition^3^ as a guiding principle: ‘a state of wellbeing in which every individual realises their own potential, can cope with the normal stresses of everyday life, can work productively and fruitfully, and is able to make a contribution to their community’.^4^^,^^5^

**Conceptual framework of DynaHEALTH:** The relationships between psychosocial factors, glycaemic health^4^^,^^5^ and healthy ageing, including a reduced risk of T2D and cardiovascular diseases, may be conceptualized in several different ways. It is an integral part of the DynaHEALTH consortium to develop these concepts and translate them into corresponding analytical study designs compatible with available data (Figure 2).

At the core of DynaHEALTH is the well-established observational and likely causal relationship between stages of disease development: firstly between adiposity and the risk of deteriorating glycaemic health and eventual T2D,^6–8^ and secondly between T2D and the risk of non-communicable diseases including stroke, coronary heart diseases, dementia and Alzheimer’s disease. All of these (pre-)clinical stages of disease development may share genetic, biological, lifestyle and psychosocial causes, but for both stages considerable knowledge gaps remain concerning action and impact on policies. We lack knowledge on identification of specific causal factors, the pathway(s) they operate through and their life-course aspects (Figure 2).

While considering these relationships, a number of hypotheses may be posed about the role of psychosocial factors throughout the life-course from the foetal period to old age.

- **Unidirectional causality hypothesis:** Adverse psychosocial factors play a causal role in the development and worsening of adiposity, or of particular types of adiposity, with different relationships to glycaemic health and risk of T2D and subsequent morbidities.
- **Pleiotropy and interaction hypothesis:** In the case of causality in such life-course pathways, it can be hypothesized that causal reactions to one or a set of psychosocial factors reflect underlying commonality influencing the clinical outcome in the life-course process. This can be analysed by exploring how the psychosocial factors may modify or contribute to the core relations between adiposity, glycaemic health, risk of T2D and the later onset of cardiovascular diseases, so that each of these relationships becomes stronger when the individuals are exposed to adverse psychosocial factors.
- **Bi-directional causality hypothesis:** The relationships between adiposity, glycaemic health, T2D risk and cardiovascular diseases are worsening one single or set of psychosocial factor(s) in such a way that vicious cycles of deterioration are promoted.
- **Critical period hypothesis:** There are specific time periods within the life-course during which psychosocial factors have a greater impact on these unhealthy ageing pathways.
- **Biological conversion hypothesis:** The effects of psychosocial factors can be ‘transformed’ into causal biological effects. In this case, we want to identify the persisting structural effects during early life and the functional mechanisms, e.g. by epigenetic regulation of gene expression or changes in the metabolite profiles.
- **Gene**–**environment hypothesis**: The genetic variation between individuals may modify the transformation of psychosocial factors into biological effects.

The impact on future public health recommendations and new technologies strongly depends on the capacity to test this set of hypotheses. This requires large statistical power or the development of a specific study design that is enabled by building sustainable and targeted datasets through consortia such as DynaHEALTH.

## Who is in the consortium?

To date, there is no single longitudinal study with a sufficient density of data and long-term follow-up to allow a life-course study of unhealthy ageing via changes in adult glycaemic health. Nonetheless, there is a wealth of somewhat scattered, prospective studies with complementary designs which can be leveraged to study the dynamic determinants of life-long glycaemic health.

**Figure 3.**
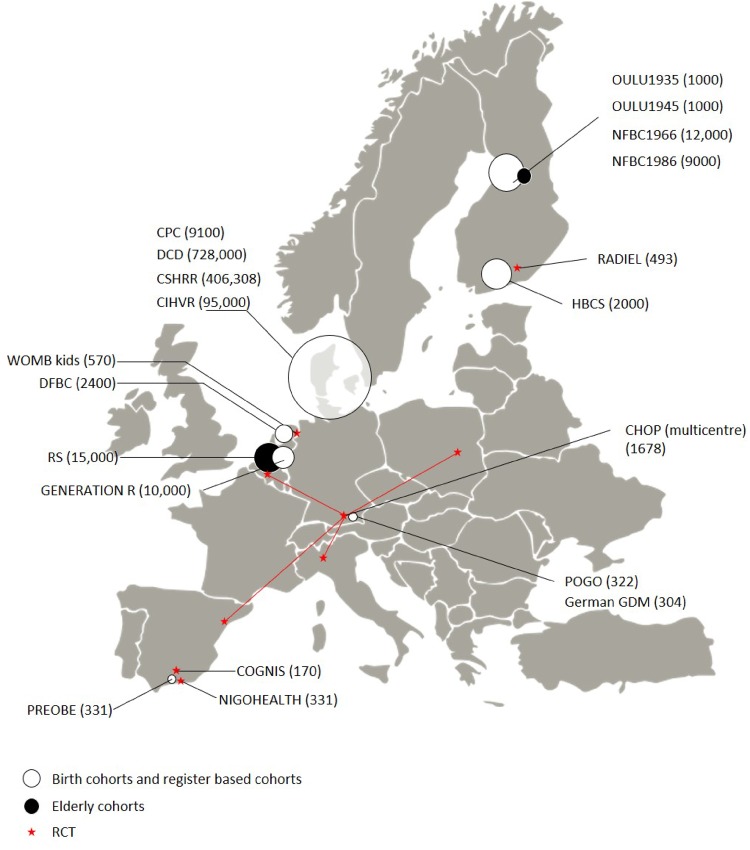
Map of studies participating in DynaHEALTH (sample size in brackets) by country. The size of the circle indicates the relative size of the study. Red arrows and stars show the participating centres of the multinational CHOP study.

**Figure 4.**
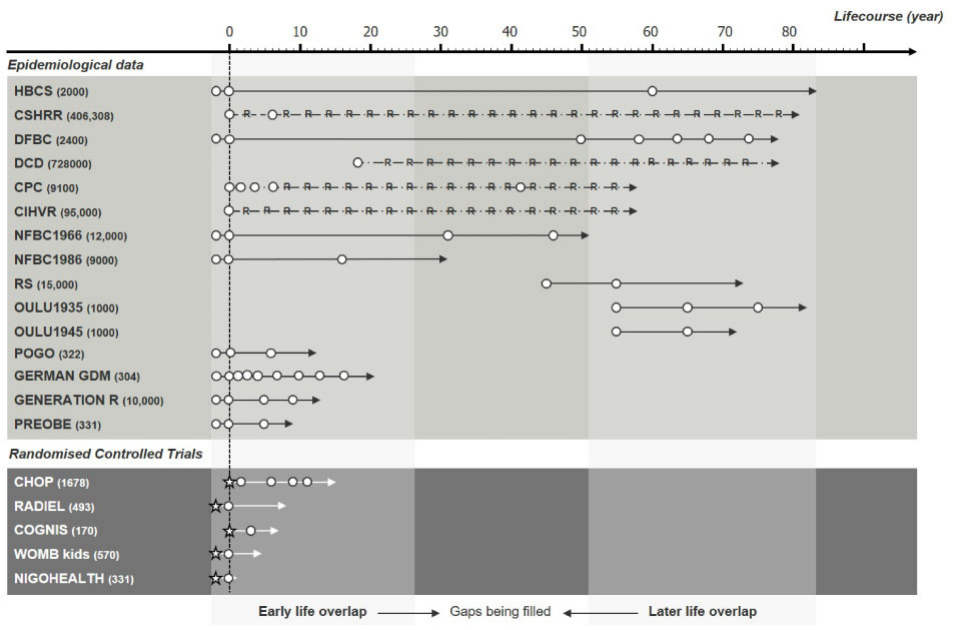
DynaHEALTH datasets by size (in brackets), type, length and timing across the life-course, from the oldest study (top) to the youngest (bottom), separately for epidemiological longitudinal data and randomized controlled trials. White circles represent follow-up points, stars represent implementation of intervention and ‘R’ represents register-based datasets. Abbreviations are given in Table 1. (Reproduced from https://www.dynahealth.eu/ with permission).

The DynaHEALTH consortium is a repertoire of human studies with longitudinal design where key variables have been inventoried for meta-analysis or triangulation of evidence to test specific epidemiological concepts. The data we are analysing are from two main types of study design: prospective longitudinal surveys and randomized controlled trials (RCT). The consortium currently consists of 20 studies (Table 1) with data from eight European Countries (Figure 3) on up to 1 368 699 participants (Figures 4). The oldest living participants are from the Helsinki Birth Cohort Study (HBCS) born in 1934 in the Finnish city of Helsinki, whereas the youngest were born in April 2018 in the NIGOHEALTH RCT in Granada, Spain. These 20 studies consist of 12 general population cohorts^9–20^ of which six have a focus on later life and ageing with data beyond 65 years of age.^9–11^^,^^17–19^ Three high-risk cohorts^21–23^ follow populations of offspring born to mothers with a risk of gestational diabetes (GDM), either via high pre-pregnancy body mass index (BMI), previous history of GDM or other known risk factors. Five RCTs focus^24–28^ on pre-conception, prenatal and early-life interventions to improve maternal health and child development.

**Table 1.** List of cohorts and randomized controlled trials participating in the DynaHEALTH consortium and their contacts

Abbreviation	Cohort full name	Contact
HBCS	Helsinki Birth Cohort Study	johan.eriksson@helsinki.fi
CSHRR	Copenhagen School Health Record Register	Jennifer.Lyn.Baker@regionh.dk
DFBC	Dutch Famine Birth Cohort	t.j.roseboom@amc.uva.nl
DCD	Danish conscription database	elme@sund.ku.dk
CPC	Copenhagen Perinatal Cohort	elme@sund.ku.dk
CIHVR	Copenhagen Infant Health Visitor Records	Jennifer.Lyn.Baker@regionh.dk
NFBC1966	Northern Finland Birth Cohort 1966	NFBCprojectcenter@oulu.fi
NFBC1986	Northern Finland Birth Cohort 1986	NFBCprojectcenter@oulu.fi
RS	Rotterdam Study	m.a.ikram@erasmusmc.nl
OULU1935	Oulu cohort study	NFBCprojectcenter@oulu.fi
OULU1945	Oulu cohort study	NFBCprojectcenter@oulu.fi
POGO **German GDM**	Postpartum Outcomes in Women with Gestational Diabetes and their Offspring	sandra.hummel@lrz.uni-muenchen.de
GENERATION R	Generation R Study	v.jaddoe@erasmusmc.nl or j.felix@erasmusmc.nl
PREOBE	Excellence Project PREOBE	ccampoy@ugr.es

Abbreviation	Randomized controlled trials full name	Contact

CHOP	Childhood Obesity Program	Berthold.Koletzko@med.uni-muenchen.de
RADIEL	Finnish Gestational Diabetes Prevention Study	johan.eriksson@helsinki.fi
COGNIS	A Neurocognitive and Immunological Study of a New Formula for Healthy Infants	Maria.Rodriguez@ordesa.es
WOMB	WOMB kids	t.j.roseboom@amc.uva.nl
NIGOHEALTH	Nutritional Intervention during Gestation and Offspring health	Ricardo.Rueda@abbott.com

This table has been adapted from https://www.dynahealth.eu/ with permission.

## How often have they been followed up?

Altogether there are 17 time periods in which data has been prospectively collected from the pre-conception period until age 80 years of age (see Figure 4 for study timelines). In addition, most follow-up time periods have data from at least two cohorts to allow replication or the use of imputation across studies. The cohort-specific descriptions of follow-ups are provided in Supplementary File 1, available as Supplementary data at *IJE* online.

## What has been measured?

The DynaHEALTH Consortium offers the potential to extend our knowledge about the childhood origin of adult metabolic diseases and focus on key exposures during pregnancy and early life and glycaemic, cardiovascular and metabolic outcomes in later life.

The consortium data harmonization policy is following a research-based focus where only variables defined in template-based analytical plans are being proposed for harmonization. The set of common harmonized data for DynaHEALTH has followed these steps adapted from Rolland *et al*.^29^

- Identification of the research questions that the harmonized data set is required to answer.
- Identification of the high-level data concepts required to answer those questions as described in the conceptual framework of DynaHEALTH.
- Assessment of data availability for data concepts.
- Inventory of sets of pre-harmonized data due to collaboration in previous consortiums.
- Development of analytical plans.
- Development of harmonized data following the FAIR data management principles.

Retrospective harmonization of data, especially the psychosocial factors, can be a costly and sometimes an impossible process. In all cases analytical procedures shall account for the source of heterogeneity in inference which is addressed by the consortium via suitable meta-analytical processes and external replication.

In many cases, the cohort studies have been able to link with national, country-specific registers. These include databases such as population, hospital, education and employment registers, health visitor records, hospital and school records. This gives us the opportunity to use objective data alongside self-reported responses. It also enables us to obtain more information than collected from the surveys, such as clinical diagnosis, hospital visits, medication use and cause of death. We also use information from these registers to conduct attrition analyses in the case of participants lost during follow-up. In summary, Tables 2 and 3 provide an overview of data available within each cohort. The co-ordinating team in DynaHEALTH has created, and regularly updates, a detailed inventory showing available data within each cohort, time points and method of collection. This allows researchers to easily identify other data sources they could use to strengthen their study results, test a hypothesis or methodology, take a life-course approach by using data at earlier or later time points or investigate historical or cultural trends. A detailed inventory of what has been measured in each cohort can also be accessed on the DynaHEALTH website (http://dynahealth.eu/).

**Table 2.** Data available in general population-based studies (√= data available, x= data not available, - =no data collection at this time point)

Indicators of interest	HBCS	CSHRR	DFBC	DCD	CPC	CIHVR	NFBC1966	NFBC1986	RS	OULU1935	OULU1945	POGO	German GDM	GenR	PREOBE
Pregnancy															
Anthropometric measures	✓	-	✓	-	✓	×	✓	✓	-	-	-	✓	✓	✓	✓
Blood samples	×	-	×	-	×	×	✓	✓	-	-	-			✓	✓
Gestational diabetes	×	-	✓	-	×	×	×	✓	-	-	-	✓	✓	✓	✓
Socio-economic indicators	✓	-	✓	-	✓	✓	✓	✓	-	-	-	✓	✓	✓	✓
Health behaviours	×	-	×	-	✓	×	✓	✓	-	-	-	✓	×	✓	✓
Childhood (birth to 12 y)															
Anthropometric measures	✓	✓	✓	-	✓	✓	✓	✓	-	-	-	✓	✓	✓	✓
Growth modelling	×	×	×	-	×	×	✓	✓	-	-	-	✓	✓	✓	✓
Blood samples	×	×	×	-	×	×	×	×	-	-	-	✓	✓	✓	✓
Developmental milestones	×	×	×	-	✓	✓	✓	✓	-	-	-	×	×	✓	✓
Early nutrition	✓	×	✓	-	✓	✓	✓	✓	-	-	-	✓	✓	✓	✓
Family lifestyle information	✓	✓	×	-	✓	×	✓	✓	-	-	-	×	×	✓	✓
Adolescence (13–18 y)															
Anthropometric measurements	-	✓	×	-	✓	-	✓	✓	-	-	-	✓	✓	✓	-
Blood samples	-	×	×	-	×	-	×	✓	-	-	-	✓	✓	✓	-
Socio-economic indicators	-	✓	×	-	✓	-	✓	✓	-	-	-	✓	✓	✓	-
Health behaviours	-	×	×	-	×	-	×	✓	-	-	-	✓	×	✓	-
Adulthood (18y +)															
Anthropometric measurements	✓	-	✓	✓	✓	-	✓	✓	✓	✓	✓	×	×	-	-
Blood samples	✓	-	✓	-	×	-	✓	✓	✓	✓	✓	×	×	-	-
Socio-economic indicators	✓	-	✓	✓	✓	-	✓	✓	✓	✓	✓	×	×	-	-
Health behaviours	✓	-	✓	-	✓	-	✓	✓	✓	✓	✓	×	×	-	-

**Table 3.** Data available or being collected for clinical trials in DynaHEALTH (√= data available, x= data not available, - =no data collection at this time point)

Indicator of interest	CHOP	RADIEL	COGNIS	WOMB	NIGOHEALTH
Pregnancy					
Anthropometric measures	✓	✓	✓	✓	✓
Blood samples	×	✓			✓
Gestational diabetes	×	✓	✓	✓	✓
Socio-economic indicators	✓	✓	✓	✓	✓
Health behaviours	✓	✓	✓	✓	✓
Childhood (birth to 12 y)					
Anthropometric measures	✓	✓	✓	✓	✓
Growth modelling	✓	×	✓	×	✓
Blood samples	✓	✓	×	✓	✓
Developmental milestones	✓	✓	✓	✓	✓
Early nutrition	✓	✓	✓	✓	✓
Family lifestyle information	✓	×	✓	✓	✓
Adolescence (13–18 y)					
Anthropometric measurements	-	-	×	-	-
Blood samples	-	-	×	-	-
Socio-economic indicators	-	-	✓	-	-
Health behaviours	-	-	✓	-	-

### Pregnancy

A wealth of data is available from early life, as 16 of the study populations were established during pregnancy or even during the pre-conception period, following a cohort design or randomized clinical intervention (Figure 4). Maternal anthropometric measures such as height and weight have been recorded at various time points throughout pregnancy and in some studies at delivery, allowing the calculation of gestational weight gain. Blood samples have been taken from the mother during pregnancy and from the umbilical cord at birth enabling measures such as glucose, insulin or cardiovascular markers to be obtained. Additionally, extraction of DNA has enabled genotyping and DNA methylation arrays. Nuclear magnetic resonance (NMR)- or mass spectrometry-based metabolomics are now also available in a number of cohorts. Questionnaires were administered in some cohorts during pregnancy to collect social and demographic data such as work-related and household information. Health behaviours included smoking and alcohol use during pregnancy.

### Childhood

Anthropometric information such as weight and height is particularly dense throughout early childhood as this was collected as part of routine practice within the health and welfare clinics in many European countries. Calculation of the BMI has allowed modelling of growth curves and enables subsequent extraction of growth traits such as age at adiposity peak and rebound, and peak weight and height velocity. In addition, the health visitor records are particularly beneficial in obtaining early-life exposures such as age of achievement of common motor and cognitive developmental milestones and breastfeeding duration. Some cohorts have also collected biological samples during this time period and more recently established studies include detailed body composition measurements such as body fat percentage and skinfold thickness. Many studies include questionnaire responses in relation to a host of lifestyle information such as dietary intake, physical condition and activity, sleep duration and quality, parental smoking and alcohol use, parental occupations and maternity leave.

### Adolescence

Height, weight and BMI are readily available for participants during adolescence across the majority of cohorts, although measurements are less regular than in childhood. Some cohorts have collected biological samples that have allowed the inclusion of epigenetic and metabolomics data in collaborative analyses. Social information has been collected by questionnaire, primarily related to the social status of the parents. However, questions have been asked of the participants about their own smoking, alcohol and drug use, how they spend their leisure time and their typical diet. In addition, some cohorts are linked to registered data on school performance.

### Adulthood and old age

Anthropometric measures are readily available at many stages of adulthood, as well as blood samples. These have been used to derive a host of indicators including common cardio-metabolic biomarkers such as glucose, insulin and lipid levels and have been used to derive epigenetic and metabolomics information. Almost all cohorts have a vast array of psychosocial variables from questionnaires and national registers. Extensive information is available on employment and work-related information such as type of occupation, hours of work, employment history and income. Educational level and occupational training of the participant and their parents is also reported in many cohorts. We can obtain a wealth of information relating to family life, such as marital status, number of children, housing situation and living environment and their changes over time. Many cohorts have collected a range of background information relating to lifestyle and health behaviours including diet, physical activity, smoking and alcohol consumption. For old-age populations especially, measures of metabolic traits and cognition have been collected.

## What has DynaHEALTH found? Key findings and publications

A full list of publications arising from the DynaHEALTH action can be found on the project website (https://www.dynahealth.eu/publications-map). DynaHEALTH aims to operationalize a data-driven approach to provide evidence for the bio-psychosocial pathways of unhealthy ageing associated with alteration of glycaemic health. This should also be examined in the context of strong health inequalities and complex transgenerational issues operating from the pre-conceptional period onwards. In support of others, we have identified several metabolic alterations in mothers with obesity and GDM in comparison with normal weight mothers, associated with offspring health, including changes in DNA methylation,^30^ gene expression^31^ and/or later metabolic outcomes.^32^^,^^33^ From an age-related perspective, DynaHEALTH research has also contributed evidence to support links between glucose metabolism, psychosocial factors and the ageing process.^34^^,^^35^

As described in the above sections it is now essential to use and model the data to explore the nature of observed associations as a pre-requisite of informed decisions for prevention, intervention and policy making.^36^ For example, we have found little evidence for causality between maternal BMI during pregnancy and the child’s risk of obesity. Rather, it is explained by genetic transmission of BMI variants interacting with a stressful, obesogenic environment.^37^ This is supported by a further study showing that risk scores based on genetic variants linked to specific biological pathways influence body fat development from early life onwards. This study found an association between a genetic risk score based on adult BMI, and BMI at adiposity peak during infancy and abdominal fat measures at the age of 6 years.^38^

Ongoing research by the consortium is supporting the joint effects of bio-psychosocial factors on glucose metabolism.^4^ We have also established an opportunity to change the trajectory of an individual from childhood adiposity to T2D in later life.^39^

## Strengths and weaknesses: how does DynaHEALTH offer a unique opportunity and what are the main challenges faced by the consortium?

DynaHEALTH exemplifies the wealth of prospective data collected in Europe that can be harnessed to enhance our understanding of healthy ageing by modelling the relationship between glycaemic health and psychosocial factors throughout the life-course. When combined, these data offer immense potential to inform future health policy in Europe. The data are organized to enable direct replication under collaborative agreements within the consortium and a number of observations can also be meta-analysed. While sample size allowing statistical power is deemed essential for robust evidence-based strategies, it is also important to combine study designs to validate findings under different statistical assumptions. DynaHEALTH’s additional strength is to include data from RCTs. Finally, the DynaHEALTH consortium includes both longitudinal birth cohorts and ageing cohorts from the same geographical location. This is the case in Northern Finland (NFBC1966/86 and Oulu 1935/45), the Rotterdam area (Rotterdam Study and Generation R Study) and in Copenhagen (CSHRR).

The critical mass of data, expertise and long-term collaboration brought together in DynaHEALTH offers a significant resource. However, the main challenge faced by the consortium is how best to combine the characteristics of the cohorts involved. The cohorts were established for their own individual purposes before being brought together under this project and the methods of data collection have thus not been standardized a priori across the consortium. Therefore, some consideration is required for the transferability of the statistical models and there are similar challenges in harmonizing the data. In addition, this is an international project and therefore there are differences between studies and countries in technology, questionnaire data and bio-specimen collection methods, terminology and diagnosis definitions, country-specific measurement techniques and ethical requirements.

However, the consortium has made significant progress in overcoming these challenges and the overarching opportunity for DynaHEALTH is that all studies provide rich data on similar key exposures and the outcome measures of interest.

Currently, there is no single cohort with data available from pre-conception to old age including information relating to both biological and psychosocial measures in relation to health outcomes. This consortium provides unique access to a number of studies ranging over different time periods encompassing different life stages, that will enable us to use a life-course approach to model the risk of poor glycaemic health and T2D, and to better understand the dynamics of how this will change in response to other factors throughout the life stages.

The collaborating cohorts include participants from eight European countries representing general, high-risk, obese and diabetic populations. This broad range of populations enables evaluation of the consistency of results and thus provides greater generalizability of consortium findings. We also have access to expert collaborators from academia and industry with expertise in life-course epidemiology, developmental biology, genetics and epigenetics, metabolomics, biostatistics, clinical nutrition, health care, brain imaging, econometrics and European policy and knowledge management.

The key value of DynaHEALTH is that we foresee the well-established, strong collaboration being used as a platform for many further efforts and continuing beyond the Horizon 2020 programme. Thus, this consortium may provide valuable assistance to investigators planning new cohort studies in terms of study design and the addition of new ideas, providing advice about data collection and management. DynaHEALTH will also offer an example of assumption-based modelling of the dynamic relations of the psychosocial and metabolic factors in the pathways of adiposity -> glycaemic health –> T2D risk –> risk of cardiovascular disease –> unhealthy ageing throughout the life course.

## Can I get hold of the data? Where can I find out more?

The project is co-ordinated by the Centre for Life Course Health Research and the Northern Finland Cohort Project Centre at the University of Oulu in Finland. Further details on DynaHEALTH are available from the website: www.dynahealth.eu. The studies are approved by the local institutional review boards. Written informed consent has been obtained for participants. There is no central repository for the data and each participating cohort has its own policy for data sharing.

The DynaHEALTH project legacy will be a collaborative action that will invite researchers with longitudinal life-course data to engage with us via the website (https://www.dynahealth.eu/contact). To ensure continuity, the relevant section of the website and a light governance will remain to bring sustainability to the consortium and support the testing of its scientific concept.

**DynaHEALTH profile in a nutshell**

- DynaHEALTH exemplifies the wealth of prospective data collected in Europe that can be harnessed to enhance our understanding of healthy ageing by modelling the relationship between glycaemic health and psychosocial factors throughout the life-course.
- DynaHEALTH includes data on approximately 1.3 million subjects within 20 cohorts in eight European countries. Collectively, data spans the life-course from pre-conception through childhood and adulthood into older age.
- Each individual study will adhere to its own protocol but generally we have repeated data collections during pregnancy and very early childhood. A number of studies have continued follow-up visits through to middle or old age. Other studies beginning in middle age have repeated measures relating to ageing.
- Data have been collected via clinical examinations including biological samples, brain scan images and questionnaire data relating to psychosocial variables. Many studies also include linkage to national registers.
- Further details are available from the DynaHEALTH website: www.dynahealth.eu

## Funding

This project received funding from the European Union’s Horizon 2020 research and innovation programme under grant agreement No 633595.

## Supplementary Material

dyz056_Supplementary_Data
