# Supplementary material for: Cohort Profile: The DynaHEALTH consortium – a European consortium for a life-course bio-psychosocial model of healthy ageing of glucose homeostasis
Source: Int J Epidemiol. 2019 Apr 10;48(4):1051–1051k. doi: 10.1093/ije/dyz056 (PMC6693805; doi:10.1093/ije/dyz056)
Supplement: dyz056_Supplementary_Data [file dyz056_supplementary_data.zip › dyz056-Suppl_data/Supplementary_Data1.docx]

***Supplementary files***

***Supplement 1: DynaHEALTH data consortium - Epidemiological studies***

**The Helsinki Birth Cohort Study (HBCS)** was established using a sample of men and women who were born at Helsinki University Central Hospital or at the Midwives’ Hospital in Helsinki, Finland between 1934 and 1944 and who attended child welfare clinics in the city of Helsinki and were still resident in Finland in 1971. This unique epidemiological cohort study includes 13,345 subjects. HBCS is a longitudinal study with data throughout the life span including prenatal life, early childhood and later life. Besides extensive epidemiological data, over 2,000 subjects have been randomly selected to attend a clinical examination. The subjects have been followed up clinically for over one decade with extensive phenotypic data available including metabolic and genetic data (genome-wide, GWAS), dietary information, other lifestyle data as well as psychological factors including personality characteristics, depression and anxiety.

**The Copenhagen School Health Records Register (CSHRR)** is a population-based cohort containing computerised information on birth weight and repeated weight and height measurements at ages 7 through 13 years on 406,308 children who were born between 1930 and 1997 and who ever attended school in the Copenhagen municipality. Until 1983, the children underwent annual health examinations by school doctors or nurses, which included measurements of height and weight. Thereafter, the children were only measured at school entry and exit unless they had special health needs. The register contains virtually every schoolchild from this time period from public and private schools. Via personal identification numbers, CSHRR is linked to a large number of national registers on vital statistics, health, economic and social conditions. In addition, the register has been linked to several Copenhagen cohorts providing information on measures of body size (body mass index (BMI) for various sub-samples of individuals later in adult life.

**The Dutch Famine Birth Cohort** is a historical cohort of originally 2,414 men and women who were born between 1 November 1943 and 28 February 1947 in the Wilhelmina Gasthuis, a local hospital in Amsterdam (15). People were included in the cohort if they were live born singletons after a pregnancy duration of at least 259 days and if a medical birth record could be retrieved. Preterm babies were thus excluded. Between 1994 and 1996, the population registry traced 2,155 (89%) of the 2,414 eligible persons. Of these, 265 had died and 199 had emigrated from the Netherlands. The population registry asked permission of eligible persons to give their address to the researchers. A total of 164 persons refused, resulting in a cohort of 1,527 at the start of the study in 1995. Since then, five sweeps of data collection have been performed, during 1995–1997, 2002–2004, 2008–2009, 2012 and 2018, including detailed phenotypic information on cardio-metabolic health, lifestyle, psychological factors as well as biosamples collected over the years.

**The Danish Conscription Database (DCD)** includes 728,159 men born between 1939 and 1959 who were examined by physicians at the conscription board from 1957 to 1984. The conscription board register cards hold a range of information on the profession, education, marital status, health, criminal records and suitability for military service of the conscript as well as information on when and where the conscript served. A few variables were selected for digitisation, namely: conscription board district, time of conscription board examination, height, weight, educational level, and intelligence test score. The DCD was established to enable studies of the influence of early physical and mental exposures on adverse health and social outcomes from a life-course perspective. Personal identification numbers have been identified for the majority of these men, thus enabling follow-up through national registers and linkages with other Danish data resources.

**The Copenhagen Perinatal Cohort (CPC)** is a prospective birth cohort study from early foetal life onwards consisting of 9,125 individuals who were born at the Copenhagen University Hospital from September 21, 1959 to December 20, 1961. Interviews about social, general medical and obstetric history were conducted before delivery at the hospital antenatal clinic and again during the first few days after birth. The mothers and their children were invited to the hospital for a follow-up examination of the children at 1, 3 and 6 years of age. For a sub-sample of the participants, school health records and conscription board records have been identified, proving information on weight and height in childhood and early adulthood. In Denmark, all young men are requested to appear at the conscription board when they turn 18 years, to be assessed for military service. At age 42 years, a questionnaire survey on general health, life-style and socio-economic status was conducted. Personal identification numbers allow for linkages with other Danish data resources. Among other things, computerised information about maternal age, BMI, gestational weight gain, smoking, and parental socio-economic status as well as birth weight and length, day-care conditions, motor function at 1 and 3 years of age and BMI throughout life (1y, 3y, 6y, school, 42y) are available.

**The Copenhagen Infant Health Visitor Records (CIHVR)** includes 95,323 births from 1959 to 1967 in the Copenhagen municipality. At the time the records were collected, the infant health visitors visited nearly all children regularly and weighed the infants at 2 weeks, 1, 2, 3, 4, 6, 9 and 12 months of age. At these free in-home visits, the health visitors also provided guidance on nutrition and baby care. The records contain information on birth weight, weight development and infant nutrition from each of the visits, parental age, parental occupation, the condition of the home, family structure, pregnancy and delivery, and childcare. Personal identification numbers enable follow-up through national registers. Electronic scans have been made of all paper records, and selected information has been computerised for around 15,500 of the infants based upon a case-cohort design.

**The Northern Finland Birth Cohort Study 1966 (NFBC1966)** study recruited all mothers in the two northernmost provinces of Finland, Oulu and Lapland with expected dates of delivery for the year 1966. The data was collected at the 157 antenatal clinics by trained local midwives. Mothers were recruited based on the calculated term and a small percentage of the births occurred towards the end of 1965 and early 1967. The gestational age was determined from the first day of the last menstrual period or, where this date was unknown, the expected term was estimated from the date of commencement of foetal movements and progress of the pregnancy. The study covered all live born and stillborn infants with a birth weight of 600 grams or more. In total 12,068 mothers were included giving birth to 12,058 live born offspring (5,889 girls and 6,169 boys, of which 314 were twins). The data collection started from antenatal records, on average at the 16^th^ gestational week, mothers filled in the questionnaires (with the help of midwives when necessary) at the 24^th^ gestational week, and the offspring were followed up prospectively by questionnaires/clinical examinations at four different time point periods; at age 1, 14, 31 and 46 years of age and by data linkage to national health and welfare registries. The latest follow-up was completed in March 2014. The database includes extensive biological and clinical disease information (e.g. GWAS, EWAS, Exome chip, metabolomics, detailed CVD, T2D and other disease measurements).

**The Northern Finland Birth Cohort Study 1986** **(NFBC1986)** recruited similarly as in NFBC1966 all women with an expected date of birth between 1.7.1985- 30.6.1986, comprising 9,362 mothers and 9,479 children (9,432 live born children). Pregnancies were followed prospectively from the first antenatal contact (10^th^– 12^th^ gestational week) including complications/diseases such as infections and hypertension, which were further confirmed from patient records, as was the neonatal outcome. There are stored serum samples from 10-12^th^ gestational week from routine health care sample collections. The children were followed-up at the ages of 6-12 months, 7-8 years, 15-16 years and the 32-year follow-up will start in 2018. At these time points, a wide range of phenotypic, lifestyle, demographic, biological and other data were gathered using questionnaires and clinical examinations (blood pressure, pulse, physical fitness (bicycle ergometry), anthropometry, skin prick tests, blood samples including DNA, fasting glucose, insulin, lipids, selected hormones, GWAS, EWAS, metabochip data). Extensive prospectively collected growth data (20 measures of height/weight, fewer head circumferences, between 0 and 16y) are available. Linkage to national registries (hospitalisation, deaths, education, medication, pensions) provides access to additional demographic and clinical data.

**The Rotterdam Study** **(RS)** is a prospective cohort study in the Ommoord district in the city of Rotterdam, the Netherlands. Participants were recruited in multiple waves: between 1989 and 1993, almost 8,000 participants aged 55 years or over were included; in 2000/2001, a little over 3,000 participants who had turned 55 or had moved into the area were added; and in 2006-2008 almost 4,000 additional participants of 45 years and over were included. From 2016 onwards, the study includes a fourth wave of participants of 40 years and over. Participants are followed up every 3 to 4 years. The database includes a vast amount of demographic and disease-related data and data on biological measures such as GWAS, EWAS, metabolomics and inflammatory markers.

**The Oulu 1935 and 1945** **(Oulu35, Oulu45)** Oulu, Finland, has conducted two studies focusing on the ageing process, starting in 1990 and 2000 for individuals born in 1935 (Oulu35) and 1945 (Oulu45) respectively. Both of these cohorts comprise approximately 1,000 individuals living in the Oulu region with data collection beginning at age 55 years until 80 years (Oulu35) and 70 years (Oulu45). The database includes detailed data on diseases, mental health, early signs of dementia and in particular data on T2D and cardiovascular health and other clinical examinations including blood samples.

**The Postpartum Outcomes in Women with Gestational Diabetes (GDM) and their Offspring (POGO)** study invited women who were screened for GDM in the Klinikum Schwabing, a former outpatient clinic in Munich, Germany, during at least one pregnancy between 1998 and 2009. Women who were diagnosed with GDM, according to the German Diabetes Association guideline received dietary counselling and treatment of GDM (diet or insulin) with repeated follow-up visits until delivery. These mothers and their offspring were invited for a single clinical visit at the clinical study centre of the Institute of Diabetes Research, Germany, between 2011 and 2015. In total, 155 mother-offspring pairs participated in the POGO study with intensive biological and other data being collected. The children's age at the time point of data collection ranged between 1 and 17 years.

**The Prospective German GDM** study recruited a total of 302 mothers diagnosed with GDM, according to the criteria of the German Diabetes Association, and their offspring across Germany between 1989 and 1999. Both, mothers and their offspring were enrolled into the study before the offspring reached 3 months of age and followed at 9 months and 2 years post-pregnancy and in 3-year intervals thereafter. During the follow-up visits, blood samples and DNA were collected. In the mothers a 75-g OGTT was performed at each visit for the detection of postpartum diabetes. Follow-up is ongoing and so far, postpartum diabetes mellitus has been diagnosed in 167 women. Demographic data that included the age at delivery, number of preceding pregnancies, gestational age, birth weight of the child, diabetes treatment during pregnancy, BMI at the first pregnancy visit, maternal smoking during pregnancy and family history of T1D or T2D were obtained shortly after delivery. In offspring, data on height and weight were collected at each follow-up visit and insulin resistance (HOMA-IR) was assessed at age 8 and 11 years.

**The Generation R Study** is a population-based prospective cohort study from foetal life until adulthood, designed to identify early determinants of growth, development and health [Kooijman et al, Eur J Epidemiol 2016]. It focuses on a broad range of outcomes, which include behaviour and cognition, growth and body composition, cardiovascular development, respiratory health, dermatology, ophthalmology, hearing, immunology and oral health, in both parents and children. The main exposures of interest are environmental, endocrine, lifestyle, nutritional and socio-demographic determinants, as well as genetic and epigenetic factors and the microbiome. Questionnaires, interviews, detailed physical and ultrasound examinations, behavioural observations, Magnetic Resonance Imaging (MRI) and biological sampling are all part of the data collection process. (Epi-)genome-wide association screens are available. A total of 9,778 mothers with a delivery date between April 2002 and January 2006 were enrolled. Response at baseline was 61% and general follow-up rates were around 80% until the age of 10 years. The follow-up at 13 years of age is currently ongoing.

**The PREOBE study** (Role of Nutrition and Maternal Genetics on the Programming of Development of Fetal Adipose Tissue) was designed as a prospective observational cohort study and included no interventions. The study recruitment was performed between 2008 and 2012, through collaboration with the San Cecilio Clinical University Hospital and the Mother-Infant University Hospital of Granada, Spain and their peripheral health centers. Pregnant women attending antenatal clinics for regular check-ups were invited to participate in the study. The inclusion criteria were: singleton pregnancy at 12–34 weeks of gestation (preferably before 20 weeks), maternal age between 18 and 45 years, no simultaneous participation in any other research study, no drug treatment, no vegan diet, and no diagnosed diseases other than obesity, overweight or gestational diabetes. In total 331 of the 474 pregnant women assessed were eligible. 21 dropped out before the visit at 34 weeks, another 5 did not attend the visit at 34 weeks (two due to preterm delivery) but remained in the study for postnatal follow-ups. Thus, 310 mother-child pairs (142 girls and 158 boys) were considered as remaining at delivery. Data collection included dietary intake, life-style habits, physical activity, anthropometry and body composition, haematological study, biochemical study (lipid and metabolic biomarkers), immune function profile related to nutritional status, neuropsychological profile, Magnetic Resonance Imaging (MRI), genetic biomarkers, and microbiological markers; all in relation to the development of the foetal adipose tissue in the first stages of life and the risk of suffering from obesity in the future. Children of the PREOBE study were followed up prospectively at different time point periods: 3, 6, 12, and 18 months, 2, 3.5, 6.5 years of age. Currently the PREOBE follow-up at 6.5 years is expected to be finished in October 2018, and follow-up of the 8-year-old children is currently ongoing.

***DynaHEALTH data consortium - Follow-up of Randomised Controlled Trials (RCTs)***

Five randomised controlled trials are included in the ***DynaHEALTH data consortium***, two of which are concerned with the effect of the lactation period and randomised offspring born to general population parents. The final three RCTs have randomised mothers according to the prenatal environment.

**The Childhood Obesity Project (CHOP)** trial is an ongoing EU-funded project. In five European countries (Belgium, Germany, Italy, Poland and Spain), 1,678 healthy singleton term-born infants were recruited. When parents decided to formula feed their infants, these were randomised and allocated to receive higher or lower protein content formula (resembling the upper and lower limits of the EU regulation in 2002) during the first year of life (N=1090). Otherwise they were included in the breastfed reference group (N=588). The CHOP study was primarily designed to analyse the effect of nutritional intervention in infancy on BMI. Infants receiving a lower protein content formula than conventional formulas showed significantly lower early weight gain and weight-for-length at 2 years, lower BMI and obesity risk at 6 years. The CHOP study offers data from early infancy (inclusion in the study was with a median age of 14 days) to pubertal age (11 years). In addition to detailed anthropometry characteristics, other lifestyle factors were assessed. Children were followed-up closely at every month during their first year of life and at 13 following time point periods i.e. 18, 24, 30, 36, 42, 48, 54, 60, 66, 72, 84, 96, 132 months of age.

**The Finnish Gestational Diabetes Prevention Study (RADIEL)** is a randomised lifestyle intervention trial carried out between the years 2008 and 2014 in the maternity hospitals of the Helsinki metropolitan area and in the South-Karelia Central Hospital (SKCH) in Lappeenranta, in South-Eastern Finland. The study recruited women at high risk for diabetes (with a history of GDM and/or a BMI ≥ 30 kg/m2) when planning pregnancy or in the first half of pregnancy. The study was designed for a primary health care setting with the main aim to assess the efficacy and cost-effectiveness of a combined diet and physical activity intervention, implemented before, during and after pregnancy, in limiting gestational weight gain, preventing GDM and later type 2 diabetes, and reducing cardiovascular disease risk factors. The first phase of the study, including a 12-month follow-up postpartum, was completed in January 2014. In the second phase, subjects in the RADIEL cohort, including mothers, fathers and children, will be followed-up until the child is 10 years of age.

**COGNIS (Neurocognitive and Immunological Study of a New Formula for Healthy Infants)** is a cohort of 170 healthy infants less than two months of age, who were exclusively or >75% fed with infant formula, recruited at the University of Granada’s San Cecilio Hospital, Spain. The cohort is randomised to receive standard infant formula, or an infant formula enriched with Milk Fat Globule Membrane (MFGM) components, long-chain Polyunsaturated Fatty Acids, synbiotics (prebiotics and probiotics: *Lactobacillus rhamnosus* and *Bifidobacterium longum subesp. infantis* CECT7210) until the age of 18 months, in a double-blind placebo controlled study. In parallel, 50 exclusively breastfed infants for at least 4 months, were followed up as a reference group. Follow-up visits were performed at 2, 3, 4, 6, 12, 18 months and 2.5 years, and is still on-going at 4 and 6 years. At each study point, cognitive development and behaviour are measured through different validated tests: i) functional measurements (fMRI, Eye Tracking, EEG/ERP), ii) neuropsychological evaluation (K-BIT, BENCI, PLON-R, Child Behaviour Check List (CBCL), EAS questionnaire). In addition, other secondary outcomes were also evaluated such as infections, anthropometry, digestive tolerance to formula, blood pressure, sleep patterns and 24 h glucose monitoring at 6 years of age.

**Women, their Offspring and iMproving lifestyle for Better cardiovascular health of both** **(WOMB project)** is the follow-up of a randomised controlled preconception lifestyle intervention trial in overweight and obese infertile couples (LIFEstyle - NTR N°: NTR1530) (Mutsaerts *et al* NEJM 2016). The study recruited obese, infertile women in the Netherlands between 2009 and 2012. During the follow up (for protocol see Van de Beek et al BMJ Open 2018) the cardiometabolic health of both women (WOMB women) and children (WOMB kids) will be assessed, as well as growth and development of the children between the ages of 3-5 yrs.

**The Nutritional Intervention during Gestation and Offspring health (NIGOHEALTH) Follow up Study** aims to investigate the potential effects of a low glycaemic index/slow digesting (LGI/SD) nutritional product in obese pregnant women, to reduce maternal glycaemia, and growth patterns and neurodevelopment in the offspring. Obese pregnant women older than 18 years, with a pre-pregnancy BMI ≥30 kg/m² and a singleton pregnancy between 15 to 16 weeks of gestation were randomised to intervention or standard of care study groups. Subjects with a medical history which is expected to alter blood glucose regulation, or taking medications that can impact blood glucose, were not included in the study. Previously diagnosed diabetes, previous gestational diabetes, systemic lupus erythematosus, anti-phospholipid syndrome, known renal disease, treated hypertension, uncontrolled hypothyroidism, cancer or bariatric surgery were also conditions of exclusion in this study. Pregnant women follow-ups were planned for 27-28 and 34-36 weeks of gestation and birth to 72 hours post-delivery. The primary variable of the study is the maternal blood glucose area under the curve (AUC) at 28 weeks of gestation with a 75g, 2h oral glucose tolerance test (OGTT); secondary variables are the neonatal body composition, ≤48 hours after birth and the maternal fasted blood glucose (FBG) at 36 weeks of gestation.

Within DynaHEALTH, babies born to mothers from the NIGOHEALTH Study are followed up in the NIGOHEALTH FOLLOW-UP Study. Follow-ups are at five different periods i.e. at ages 4, 6, 9, 12 and 18 months of age, to assess the impact of blood glucose levels, body composition and body mass index (BMI) of obese pregnant mothers on their offspring’s growth, body composition, and neurocognitive and behaviour development. Follow-up and completion is expected during September 2019.
