# Supplementary material for: Cohort Profile: The DynaHEALTH consortium – a European consortium for a life-course bio-psychosocial model of healthy ageing of glucose homeostasis
Source: Int J Epidemiol. 2019 Apr 10;48(4):1051–1051k. doi: 10.1093/ije/dyz056 (PMC6693805; doi:10.1093/ije/dyz056)
Supplement: dyz056_Supplementary_Data [file dyz056_supplementary_data.zip › dyz056-Suppl_data/Supplementary_Data2.docx]

**Supplement 2 – additional cohort-specific acknowledgment and funding statements**

The study is fully funded by DynaHEALTH via the European Union’s Horizon 2020 research and innovation programme under grant agreement No 633595. However, the following section acknowledges previous funding and efforts allowing us to propose this data profile.

**HBCS**

HBCS has been supported by grants from British Heart Foundation, NIH, Finska Läkaresällskapet, the Finnish Special Governmental Subsidy for Health Sciences, Academy of Finland, Samfundet Folkhälsan, Liv och Hälsa, Juho Vainio Foundation, Yrjö Jahnsson Foundation, The Diabetes Research Foundation, Finnish Foundation for Cardiovascular Research, the Signe and Ane Gyllenberg Foundation, the Academy of Finland (grants no. 129369, 129907, 135072, 129255 and 126775). EU FP7 (DORIAN) project number 278603, and EU Horizon 2020 (DynaHealth) project number 633595.

**NFBC 1966 and 1986**

We thank Professor Paula Rantakallio (launch of NFBC1966 and initial data collection). We gratefully acknowledge the contributions of the participants in the Northern Finland Birth Cohort 1966 study and the Northern Finland Birth Cohort 1986. We also thank all the field workers and laboratory personnel for their efforts.

**Funding statements**

NFBC1966 received financial support from University of Oulu Grant no. 65354, Oulu University Hospital Grant no. 2/97, 8/97, Ministry of Health and Social Affairs Grant no. 23/251/97, 160/97, 190/97, National Institute for Health and Welfare, Helsinki Grant no. 54121, Regional Institute of Occupational Health, Oulu, Finland Grant no. 50621, 54231. NFBC1986 received financial support from EU QLG1-CT-2000-01643 (EUROBLCS) Grant no. E51560, NorFA Grant no. 731, 20056, 30167, USA / NIHH 2000 G DF682 Grant no. 50945. MW was supported by the European Union’s Horizon 2020 research and innovation program under grant agreement No 633212. MRJ and SS are supported by H2020-633595 DynaHEALTH action and academy of Finland EGEA-project (285547).

**The Danish Conscription Database (DCD)**

We thank Kaare Christensen, Drude Molbo, Erik L. Mortensen, and Merete Osler who together with Thorkild I.A. Sørensen established the Danish Conscription Database.

**The Copenhagen Perinatal Cohort (CPC)**

We are grateful for the collection of the Copenhagen Perinatal Cohort data led by the late Drs Aage Willumsen and Bengt Zachau-Christiansen

**The Copenhagen Infant Health Visitor Records (CIHVR)**

The CIHVR was established by the former Institute of Preventive Medicine (now the Center for Clinical Research and Prevention). It was built in collaboration with the University of Southern Denmark and the records were lent out by the Copenhagen City Archives in Denmark. We thank the nurses from the Copenhagen Municipality for their work with the infants.

**The Copenhagen School Health Records Register (CSHRR)**

The CSHRR was established by the former Institute of Preventive Medicine (now the Center for Clinical Research and Prevention). It was built in collaboration with the Copenhagen City Archives in Denmark. We thank the doctors and nurses from the Copenhagen Municipality School Health Services for their work with the children.

**PREOBE** was funded by the Regional Ministry of Innovation and Science. Junta de Andalucía Excellence Project (P06-CTS-02341); Spanish Ministry of Education (Grant no. SB2010-0025); Spanish Ministry of Economy and Competitiveness (BFU2012-40254-C03-01). Further support was received from Abbott Laboratories, Granada, Spain.

**Generation R Study**

The Generation R Study is conducted by the Erasmus Medical Center in close collaboration with the School of Law and Faculty of Social Sciences of the Erasmus University Rotterdam, the Municipal Health Service Rotterdam area, Rotterdam, the Rotterdam Homecare Foundation, Rotterdam and the Stichting Trombosedienst & Artsenlaboratorium Rijnmond (STAR-MDC), Rotterdam. We gratefully acknowledge the contribution of children and parents, general practitioners, hospitals, midwives and pharmacies in Rotterdam. The study protocol was approved by the Medical Ethical Committee of the Erasmus MC, Rotterdam. Written informed consent was obtained for all participants.

**Funding**

The general design of the Generation R Study is made possible by financial support from Erasmus MC, Rotterdam, the Erasmus University Rotterdam, the Netherlands Organization of Scientific Research (NWO), the Netherlands Organization for Health Research and Development (ZonMw), the Ministry of Health, Welfare and Sport and the Ministry of Youth and Families. VWJ received an additional grant from the Netherlands Organization for Health Research and Development (VIDI 016.136.361) and a Consolidator Grant from the European Research Council (ERC-301 2014-CoG-648916).

**CHOP**

We thank the participating families and all project partners for their enthusiastic support of the project.

The European Childhood Obesity Trial Study Group: Philippe Goyens, Clotilde Carlier, Joana Hoyos, Pascale Poncelet, Elena Dain (Université Libre de Bruxelles –Brusselles, Belgium); Françoise Martin, Annick Xhonneux, Jean-Paul Langhendries, Jean-Noel Van Hees (CHC St Vincent –Liège-Rocourt, Belgium); Ricardo Closa-Monasterolo, Joaquin Escribano, Veronica Luque, Georgina Mendez, Natalia Ferre, Marta Zaragoza-Jordana (Universitat Rovira i Virgili, Institut d’Investigació Sanitaria Pere Virgili, Taragona, Spain); Marcello Giovannini, Enrica Riva, Carlo Agostoni, Silvia Scaglioni, Elvira Verduci, Fiammetta Vecchi, Alice Re Dionigi (University of Milano, Milano, Italy); Jerzy Socha, Piotr Socha (Children’s Memorial Health Institute, Department of Gastroenterology, Hepatology and Immunology, Warsaw, Poland); Anna Dobrzańska, Dariusz Gruszfeld (Children’s Memorial Health Institute, Neonatal Intensive Care Unit, Warsaw, Poland); Anna Stolarczyk, Agnieszka Kowalik (Children’s Memorial Health Institute, Department of Pediatrics, Warsaw, Poland); Roman Janas, Ewa Pietraszek (Children’s Memorial Health Institute, Diagnostic Laboratory, Warsaw, Poland); Emmanuel Perrin (Danone Research Centre for Specialized Nutrition, Schiphol, The Netherlands); Rüdiger von Kries (Division of Pediatric Epidemiology, Institute of Social Pediatrics and Adolescent Medicine, Ludwig Maximilians University of Munich, Munich, Germany); Helfried Groebe, Anna Reith, Renate Hofmann (Klinikum Nurnberg Sued, Nurnberg, Germany); Berthold Koletzko, Veit Grote, Martina Totzauer, Peter Rzehak, Sonia Schiess, Jeannette Beyer, Michaela Fritsch, Uschi Handel, Ingrid Pawellek, Sabine Verwied-Jorky, Iris Hannibal, Hans Demmelmair, Gudrun Haile, Melissa Theurich, Phillipp Schwarzfischer (Division of Nutritional Medicine and Metabolism, Dr. von Hauner Childrens Hospital, University of Munich Medical Centre, Munich, Germany)

**Funding**

The studies reported herein have been carried out with partial financial support from the Commission of the European Community, specific RTD Programme "Quality of Life and Management of Living Resources", within the European Union's Seventh Framework Programme (FP7/2007-2013), project EarlyNutrition under grant agreement no. 289346, the EU H2020 project PHC-2014-DynaHEALTH under grant no. 633595 and the European Research Council Advanced Grant META-GROWTH (ERC-2012-AdG – no.322605). This manuscript does not necessarily reflect the views of the Commission and in no way anticipates the future policy in this area.
